# Identification of Thalidomide-Specific Transcriptomics and Proteomics Signatures during Differentiation of Human Embryonic Stem Cells

**DOI:** 10.1371/journal.pone.0044228

**Published:** 2012-08-28

**Authors:** Kesavan Meganathan, Smita Jagtap, Vilas Wagh, Johannes Winkler, John Antonydas Gaspar, Diana Hildebrand, Maria Trusch, Karola Lehmann, Jürgen Hescheler, Hartmut Schlüter, Agapios Sachinidis

**Affiliations:** 1 Center of Physiology and Pathophysiology, Institute of Neurophysiology, Cologne, Germany; 2 Institute of Clinical Chemistry, University Medical Center, Hamburg-Eppendorf, Hamburg, Germany; 3 Institut of Organic Chemistry, Mass Spectrometry Group, Universität Hamburg, Hamburg, Germany; 4 Proteome Factory AG, Berlin, Germany; University of Cincinnati, United States of America

## Abstract

Embryonic development can be partially recapitulated *in vitro* by differentiating human embryonic stem cells (hESCs). Thalidomide is a developmental toxicant *in vivo* and acts in a species-dependent manner. Besides its therapeutic value, thalidomide also serves as a prototypical model to study teratogenecity. Although many *in vivo* and *in vitro* platforms have demonstrated its toxicity, only a few test systems accurately reflect human physiology. We used global gene expression and proteomics profiling (two dimensional electrophoresis (2DE) coupled with Tandem Mass spectrometry) to demonstrate hESC differentiation and thalidomide embryotoxicity/teratogenecity with clinically relevant dose(s). Proteome analysis showed loss of POU5F1 regulatory proteins PKM2 and RBM14 and an over expression of proteins involved in neuronal development (such as PAK2, PAFAH1B2 and PAFAH1B3) after 14 days of differentiation. The genomic and proteomic expression pattern demonstrated differential expression of limb, heart and embryonic development related transcription factors and biological processes. Moreover, this study uncovered novel possible mechanisms, such as the inhibition of RANBP1, that participate in the nucleocytoplasmic trafficking of proteins and inhibition of glutathione transferases (GSTA1, GSTA2), that protect the cell from secondary oxidative stress. As a proof of principle, we demonstrated that a combination of transcriptomics and proteomics, along with consistent differentiation of hESCs, enabled the detection of canonical and novel teratogenic intracellular mechanisms of thalidomide.

## Introduction

Traditional approaches to toxicological testing typically involves exposure of chemicals to large numbers of animals during the crucial period of organ development and further investigations of foetuses for visceral and skeletal developments, these approaches are expensive and time consuming [1]–[4]. In order to provide cost-efficient and high throughput methods, a multitude of *in vitro* test systems have been proposed to assess the developmental toxicity of candidate drugs and environmental toxicants in the past 20 years. These platforms include primary *in vitro* cell cultures and *ex vivo* models using embryo cultures [5] Embryonic stem cells (ESCs) have the unique potential to differentiate into all somatic cell types. In this context, a mouse ESC test originally covering the three end points for predicting teratogenicity (ESC cytotoxicity, fibroblasts cytotoxicity, and the inhibition of ESC differentiation into cardiomyocytes) has been initiated [6], [7] . Although these methods are able to predict toxicity of the drugs, hESC were introduced for toxicity testing in order to better reflect the human physiology and to avoid interspecies differences [8].

The embryotoxic drug thalidomide (Contergan) was launched in 1957 in Germany and was subsequently withdrawn in November 1961 after its teratogenic effects in humans were recognised. The clinical evidences showed that thalidomide causes various phenotypic malformations such as limb, ear, ocular, kidney, heart and gastrointestinal deformities (Reviewed in [9], [10]). To determine the teratogenic potential of thalidomide, various *in vivo* assessments were applied to different animal species based on traditional clinical and histopathological measurements. Thalidomide induced distinct developmental adverse effects in different animals such as dogs, rats, mice and rabbits [11], [12]. Moreover, congenital malformations induced by thalidomide were prominently found in rabbits where as in rats moderate effects were found and in mice no significant foetal changes were observed [11], [13], [14]. Transcriptional profiling of cynomolgus monkeys at 26–28 days of gestation, observed limb defects and down-regulation of vasculature development-related transcripts [15].

To unravel the molecular mechanisms of thalidomide in embryonic development various *in vitro* models has been utilised, predominantly primary cells from non human origin and many hypotheses or mechanisms have been proposed for thalidomide teratogenecity [16]–[19]. It was demonstrated that generation of reactive oxidative species (ROS), oxidative DNA damage and perturbation of intra cellular signalling such as FGF, WNT and AKT are the causes of thalidomide induced limb deformities [9], [20]. The generation of ROS leads to oxidation or alteration of glutathione content. This is crucial for detoxification of cells, especially oxidative stress conditions and regular embryonic development [21]. Although these studies described the required toxic dose and perturbations of organ development, these results cannot necessarily be extrapolated to other species or humans due to the known species-specificity of thalidomide [22]. Therefore, a consistent and predictive developmental toxicity model based on hESCs requires an in-depth insight into the molecular mechanisms that explain the adverse developmental potential of a drug in the concentration range applied under *in vivo* conditions. Omics approaches using ESCs as a model were proposed as an innovative way of drug safety testing (reviewed in [23]).

Multilineage differentiation of human embryonic stem cells (hESCs) can partially reproduce early human embryonic development [24]. Therefore, hESCs are a suitable tool to assess toxicant profiles to understand and predict the damage caused by potential therapeutic agents. To test the applicability of the hESCs as a proof of principle human *in vitro* embryotoxicity model, we applied a well-defined differentiation protocol. We studied thalidomide, a highly investigated *in vivo* developmental toxicant, at different concentrations with a combination of sensitive transcriptomics and proteomics approaches. Here, we demonstrated that low concentrations of thalidomide, normally observed in the plasma under *in vivo* conditions, induced the perturbation of genes and proteins participating in limb, heart development and oxidative stress-mediated nuclear cytoplasmic protein transport.

## Methods

### Ethics Statement

Experiments were approved by the governmental animal care and use office (Landesamt für Natur, Umwelt und Verbraucherschutz Nordrhein- Westfalen, Recklinghausen, Germany, Protocol No. 8.84–02.05.30.11.030) and were in accordance with the German Animal Welfare Act.

### hESCs culture conditions and differentiation

**Figure 1:**
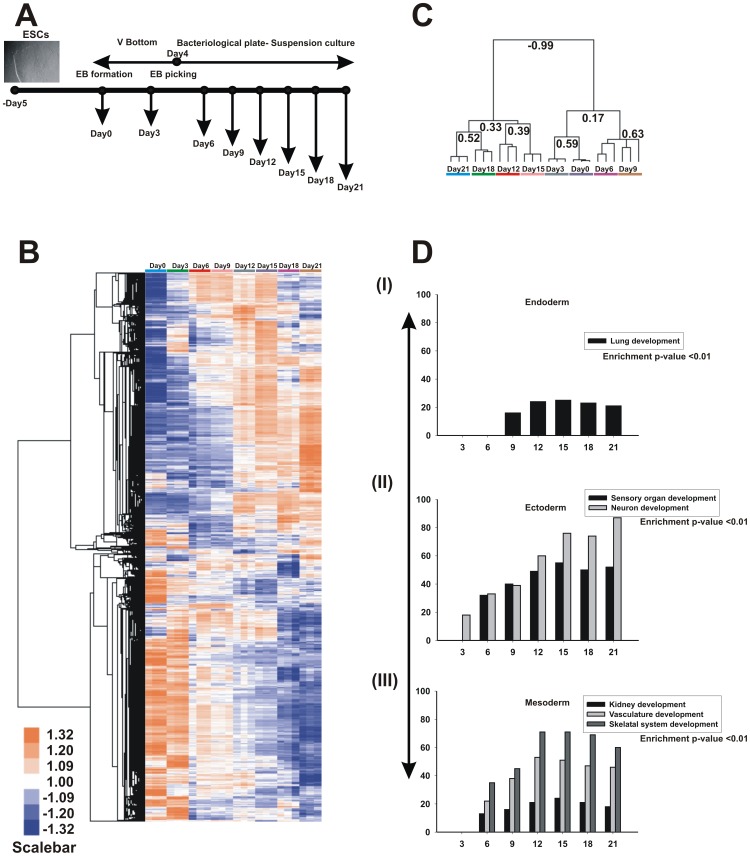
A temporal gene expression profiling of hESC differentiation. **A**, Experimental representation of the time kinetic multilineage differentiation. **B**, An unsupervised hierarchical clustering analysis demonstrates that a temporal gene expression pattern changes during differentiation and the major changes were observed between days 12 to 14. Three independent biological experiments were performed for statistical analysis (p≤0.05). **C**, The dendrogams pattern of array cluster showed that a two major negative correlation for day 0 to 9 and 12 to 21 differentiation. **D**, The selected germ layer lineage specific BP (Enrichment p≤0.01) for up-regulated transcripts demonstrated that the numbers of genes are optimal between days 12 and 15. The figure represents (i) endoderm-specific, ii) ectoderm-specific and iii) mesoderm-specific BP. The x-axis represents the days of differentiation, and the y-axis represents number of transcripts.

H9 hESCs (WiCell, Madison, WI, USA) were cultured in DMEM-F12, 20% KO serum replacement, 1% non-essential amino acids, penicillin (100 units/ml), streptomycin (100 µg/ml) and 0.1 mM β-mercaptoethanol supplemented with 4 ng/ml basic fibroblast growth factor (bFGF). The cells were passaged with mechanical dissociation on irradiated mouse embryonic fibroblasts (MEF) which were prepared as described previously [25]. Prior to differentiation, the cells were maintained for five days in 60-mm tissue culture plates (Nunc, Langenselbold, Germany) coated with a hESC-qualified matrix (BD Biosciences, California, USA) in mTESR medium (Stem Cell Technologies). For the time kinetic multilineage differentiation, embryoid bodies (EBs) were prepared as described previously [24] with minor changes (60 to 70 clumps were added instead of 50 to 60), and the EBs were maintained for 21 days on a horizontal shaker (Figure 1A). For the dose-response analysis, thalidomide (0.01 µM, 0.1 µM, 1 µM, 10 µM, or 70 µM) (Sigma, Steinheim, Germany) was added to the medium from day 0 (undifferentiated ESCs) to day 14. Equal volumes of the highest solvent concentration were added as controls. For 2DE analysis, a 10 µM thalidomide concentration was used. On alternating days, the medium was replaced with fresh medium containing the drug. All of the experiments for microarray and 2DE analysis were performed as three independent (n = 3) biological replicates.

### Microarray labelling and hybridisation

To isolate total RNA for time kinetic analysis, samples were collected from undifferentiated hESCs and differentiated EBs for every 3 days from day 3 to day 21 (Figure 1A). For the dose-response analysis, samples were procured from hESCs, 14-day-old EBs treated with thalidomide (0.01 µM, 0.1 µM, 1 µM, 10 µM, or 70 µM) and the controls. The samples were homogenised in Trizol (Invitrogen, Darmstadt, Germany), and RNA was extracted using Trizol and CHCl_3_ (Sigma, Steinheim, Germany). The total RNA was purified using an RNeasy mini kit (Qiagen, Hilden, Germany) according to the manufacturer's instructions. A Nanodrop (ND-1000, Thermo-Fisher, Langenselbold, Germany) was used to measure the RNA quantity. The integrity of the RNA was confirmed using denaturing agarose gel electrophoresis. For the transcriptional profiling of human cells, the Affymetrix Human Genome U133 plus 2.0 arrays were used. For RNA amplification, 100 ng total RNA was used with a Genechip 3′ IVT Express Kit (Affymetrix, High Wycombe, United Kingdom) according to the manufacturer's instructions. For the aRNA purification, magnetic beads were used, and 15 µg of aRNA were fragmented. Fragmented aRNA (12.5 µg) was hybridised to microarrays in the Genechip Hybridisation Oven-645 (Affymetrix, High Wycombe, United Kingdom). The Affymetrix HWS kit was used for staining and washing with a Genechip Fluidics Station-450 according to the manufacturer's instructions. The stained arrays were scanned with an Affymetrix Gene-Chip Scanner-3000-7G, and the quality control analysis was performed with Affymetrix GCOS software.

### Data analysis and statistical procedures

Background correction, summarisation and normalisation were performed with a Robust Multi-array Analysis. The raw dataset was normalised with the Quantile normalisation method (executable with a R-affy package) performed at the probe feature level. Probe sets having a signal of ≤6 (log2 scale), in any one of the conditions out of 21, were chosen for statistical analysis. The differentially expressed genes (DEG) were described by a linear model implementing R- LIMMA packages (Linear Models for Microarray Data). A one-way ANOVA calculation was performed, considering either the treatment or the days of differentiation as a factor. A pairwise comparison was performed, and the p-values of the scores of the moderated t-test calculation were used further for statistical correction. Benjamini-Hochberg method was used to adjust the raw p-values to control the false discovery rate. Differentially expressed transcripts were filtered with a FDR-controlled p-value of ≤0.05 (95% confidence interval) along with fold change values at a threshold value of ≥±2. To find the dose-response or time kinetic effects across the dose or time, DEG were derived from F statistics. The distances of the samples were calculated using a Spearman Rank correlation. A principal component (PC) analysis was performed using the stats package in R with a prcomp function. The "x" attribute of the prcomp object was used to generate three-dimensional scatter plots.

### Gene expression and functional annotation analysis

To find the common DEG for thalidomide treatment an intersect analysis were performed. Briefly, the DEG from the temporal gene expression was used to calculate probable intersects among them. The counts are represented in the plot as number of genes. For temporal and dose response hierarchical clustering, the intensity values of the DEG from F statistics were used for uncentred correlation and average linkage clustering and visualised using Java Treeview. Transcripts were filtered with gene vector 0.2 and resulting 18567 transcripts for temporal and for dose response experiments 1076 genes from 70 µM were used for cluster analysis. The red colour represents up-regulated transcripts, and the blue represents down-regulated transcripts. To investigate the DEG, functional annotation and gene ontology (GO) clustering, the Database for Annotation, Visualisation and Integrated Discovery (DAVID) was used [26]. These analysis explore the biological processes (BP) related to the transcripts, which were differentially regulated in time kinetic and dose-response analysis. To increase stringency, GO categories with less than five transcripts were eliminated and filtered further based on the EASE score enrichment p-value (P≤0.01) and clustering stringency at the medium level as per DAVID.

### Quantitative real time PCR

An independent dose-response experiment was performed with thalidomide (1 µM, 10 µM and 70 µM), and the total RNA was extracted as described above. cDNA synthesis was performed with the Super Script Vilo cDNA synthesis kit (Invitrogen, Darmstadt, Germany) using 2 µg of total RNA as starting material. The cDNA was diluted 20 times with nuclease-free water, and 100 ng was used as a template for PCR. The primer design was performed using Primer3, or the sequences were obtained online (http://www.origene.com/). The assay was accomplished using the Platinum® SYBR® Green qPCR SuperMix (Invitrogen, Darmstadt, Germany) with 0.5 µM of the primer using the Applied Biosystems 7500 FAST cycler. The primer sequences are listed in Table S1. All of the gene expression values were normalised to the reference β-actin. The mRNA expression levels were represented as a relative quantification.

### Immunostaining

From an independent dose-response experiment on day 12 (1 µM, 10 µM, and 70 µM), EBs were dissociated with 0.05% trypsin for 10 minutes, and single cells were seeded on a fibronectin coated coverslip. On day 14, the cells were fixed with ice-cold methanol for 10 minutes at −20°C. The cells were then rehydrated three times (10 min each) using phosphate buffered saline with Ca^++^ and Mg^++^ (PBS). The samples were blocked with 5% bovine serum albumin (BSA) for 60 minutes and incubated with the primary antibody (β-Catenin (BD Biosciences, California, USA), GSTA1, GSTA2 (Abgent Europe, United kingdom), RANBP1 (Calbiochem, Darmstadt, Germany) overnight. The samples were then washed three times with PBS for 15 minutes and incubated with the respective secondary antibody conjugated to Alexa Fluor 488/594 (Millipore, Schwalbach, Germany) for 60 minutes. The samples were washed three times with PBS for 15 minutes and then mounted with Prolong Gold anti-fade with DAPI (Invitrogen, Darmstadt, Germany). The samples were observed with a Zeiss Axiovert 200 fluorescence microscope (Carl Zeiss Microscopy, Oberkochen, Germany).

### Two-dimensional gel electrophoresis (2DE) and Proteomics analysis

The frozen cell pellets were dissolved in a sample buffer for isoelectric focusing (6 M urea, 3 M thiourea, 2% ampholytes, and 70 mM DTT). The proteins were separated by 2-DE according to a protocol previously used by Klose and Kobalz [27] with modifications by Zimny-Arndt [28]. The proteins (100 µg total protein amount) were anodically loaded and focused in the first dimension (isoelectric focusing) at 8,800 Vh in a gradient between pI 3 and pI 11. The isoelectric focusing gel was applied on top of a 15% SDS–PAGE acrylamide gel (gel size 20 cm×30 cm; thickness of 1.0) to separate the proteins by size in the second dimension. The SDS-PAGE gels were fixed in 50% ethanol and 10% acetic acid for approximately 16 hours. The gels were silver stained with a mass spectrometry compatible silver staining kit (Proteome Factory PS-2001). The stained gel images were aligned using DECODON Delta2D software (http://www.decodon.com) within the biological replicates. To derive the differentiation markers, 14 day old EB gels were aligned with hESCs. The thalidomide-specific protein treatment gels were aligned with the untreated samples using warp transformations. For spot detection and quantification, the fusion gel was prepared from the respective replicates using the spot preservation and image fusion algorithm from Delta2D. The spot pattern was transferred from the fused image to all of the original images. A typical 2D image is shown in Figure S2. To derive the significantly regulated protein spots, the t-test was applied with a statistical value of p≤0.01.

### Tryptic digestion

The protocol for tryptic digestion was based on a protocol of Bertinetti et al with some modifications. 2DE-spots were picked and incubated with a shrinking solution consisting of 50 mM ammonium bicarbonate (Sigma Aldrich, Germany) in a 60% acetonitrile/water solution (Lichrosolv, hyper grade, Merck, Germany) for 5 minutes. The shrinking buffer was removed, and the gel was incubated with a swelling buffer containing 100 mM ammonium bicarbonate. Each gel band was placed into a 10-µl pipette tip that was then placed in a shortened 1000-µl tip and inserted into a borosilicate vial (CS-Chromatographie Service, Germany). This apparatus was placed in a 1.5 ml tube and centrifuged for 5 min at 14,000 rpm (Centrifuge 5415C, Eppendorf, Germany). The obtained gel slurry was dried by evaporation (RC10, Thermo Fisher Scientific, USA), and the proteins were digested overnight at 37°C after the addition of 20 µl trypsin (final concentration 10 ng/µl, dissolved in trypsin resuspension buffer, Promega, USA) to the gel. The reaction was stopped by the addition of a 5% formic acid and water solution (final concentration 0.1%), and the supernatant was taken from the gel slurry. The peptides were extracted twice by the addition of 10 µl of a solvent containing 65% acetonitrile with 35% formic acid (5% v/v in water) and 10 µl of 100% acetonitrile, respectively. The resulting peptide fractions were evaporated until they were completely dry. The fractions were then resolved in 10 µl of solvent (5% acetonitrile and 0.2% formic acid in water) prior to LC-MS analysis.

### Protein identification by LC-MS-Analysis

Identification was performed on an Agilent 1100 LC/MSD-trap XCT series system. The electrospray ionization system was the Chip Cube system using a ProtID-Chip-43 (Agilent Technologies). Sample loading from the microtiter plate onto the enrichment column was performed at a flow rate set to 4 µl/min with two mobile phases at a ratio of 98:2 (mobile phase A: 0.2% FA in H2O; mobile phase B: 100% ACN). LC gradient was delivered with a flow rate of 500 nl/min. Tryptic peptides were eluted from the reversed-phase column into the mass spectrometer using a linear gradient elution of 2–30% B in 20 min. For MS experiments, the following mode and tuning parameters were used: Scan range: 300–2,000 m/z, polarity: positive, capillary voltage: −1,800 V, flow and temperature of the drying gas were 4 l/min and 325°C. The MS/MS experiments were carried out in auto MS/MS mode using a 4 Da window for precursor ion selection. After 3 MS/MS spectra, the precursor ions were actively excluded from fragmentation for at least 1 min. The generic files for database searching were generated by Data Analysis software version 4.0. For precursor ion selection, a threshold of 5 S/N was applied and the absolute number of compounds was restricted to 1,000 per MS/MS experiment (according to (F-23) with modifications. Protein identification was performed by Mascot software (Version 2.3) using the UniProtKB database (http://www.uniprot.org/)

### Western blotting

For western blot analysis, the samples procured from the undifferentiated hESC, thalidomide treated (1 µM, 10 µM, and 70 µM) and untreated EBs at day 14 was used (An independent experiment was performed for validation). The samples were washed with PBS and lysed with ProteoJET (Fermentas, Germany) mammalian cell lysis reagent. The clarified samples were quantitated with the Bradford reagent (Sigma, Steinheim, Germany). The protein (30 µg) was separated using either 10% or 12% SDS-PAGE gels and then transferred to a PVDF membrane. The membranes were blocked with 5% BSA at room temperature and incubated with RANBP1 (Calbiochem, Darmstadt, Germany), KPNA2, DDAH2 (Abcam, Cambridge, UK), GSTA1, GSTA2, GATA6 and HAND1 (Abgent Europe, United kingdom), β-Catenin (BD Biosciences, California, USA) primary antibodies for overnight at 4°C on an orbital shaker. After washing with PBST (PBS with 0.1% Tween-20), the membranes were incubated with the respective secondary antibody (labelled with HRP) for 1 hour at room temperature. The antibodies were detected using the ECL Western Detection System (Fisher Scientific GmbH, Schwerte, Germany).

## Results

### Time kinetic analysis of hESC differentiation

A global transcriptomic analysis for time kinetic differentiation was performed to determine the optimal differentiation time point that showed the highest expression levels of developmental genes (Figure 1A). A total of 18567 DEG, derived from the ‘F statistics’ (Table S2A), were used for hierarchical cluster analysis. The gene cluster demonstrated that the major up-regulated transcripts occurring up to day 9 were repressed after day 12. The over expressed genes between days 12 and 15 were consistently induced up to day 21 (Figure 1B). Furthermore, the gene array dendrograms results showed that days 0 to 9 and days 12 to 15 were negatively correlated (−0.99) (Figure 1C). To find the common genes between the time points, intersect gene analysis was performed (as mentioned in methods). A consistent expression of up-regulated genes were observed from days 12 to 21 and compared to days 6 to 12 (selected intersects are shown in Figure S1, and the complete list given in Table S2B). To derive the biological rational for the up-regulated transcripts (Table S2C to S2I), DAVID GO analysis was performed. The BP for various organ developments (represented by the endoderm, ectoderm and mesoderm lineage) demonstrated that maximum numbers of genes were regulated between days 12 to 15 of differentiation (Figure 1D). These results confirm that differentiation for 12 to 15 days can demonstrate the embryonic development with optimal gene expression of development related genes.

### hESC differentiation for 14 days can demonstrates embryonic development

**Figure 2:**
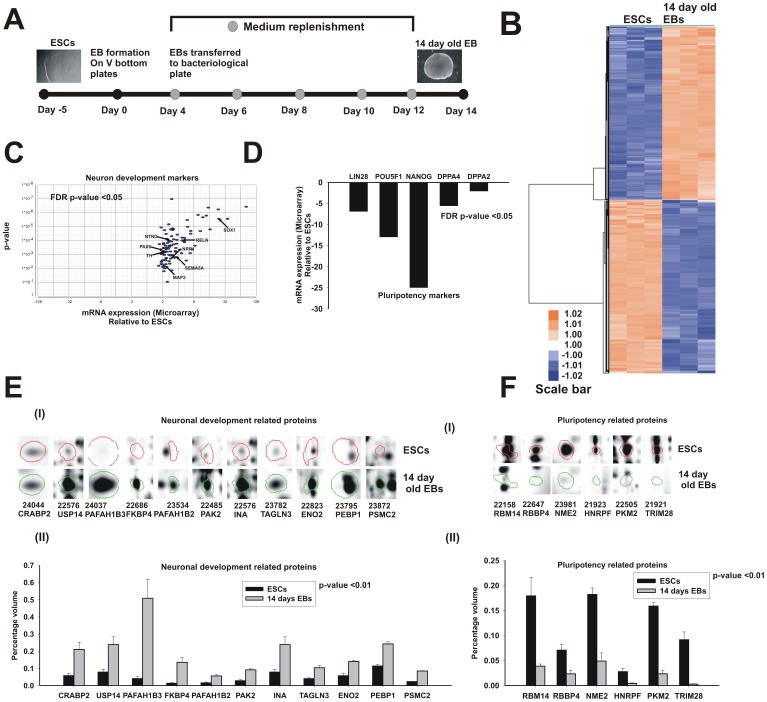
Enrichment of embryonic development associated genes in 14 days of hESC differentiation. **A**, A schematic representation of the 14 days hESC differentiation. **B**, An unsupervised hierarchical clustering analysis of DEG in hESC differentiation showed two distinct clusters for up and down-regulated genes. **C**, The over expressed neuron development BP (from Table S4) encompasses 86 genes and their gene expression pattern showed in volcano plot. In x-axis represents fold change and y-axis showed FDR-controlled p-values (p≤0.05). **D**, The pluripotency markers were significantly downregulated (as shown by microarray) after 14 days of differentiation. **E**, The mass spectrometry analysis of regulated proteins unravelled the up-regulated spots for neuronal development related proteins and (**F**) the down-regulated spots for pluripotency regulators. The differentially regulated proteins (n = 3), (p≤0.01) are shown with their corresponding spot ID and UniProt ID (E (I), F (I)). Their expression patterns are represented as a percentage of volume (E (II), F (II)). The error bar represents SEM from 3 independent biological replicates.

In order to uncover multiple embryonic development perturbations in presence of potential toxicants, it is critical to determine the optimal time point of hESC differentiation. From time kinetic experiment it was observed that between 12–15 days embryonic development related genes and biological processes were highly up-regulated, suggesting 14 days hESC differentiation as an optimal period for further study. The differentiation scheme is briefly described in Figure 2A. Comparison between ESCs after a 14-day differentiation in microarray analysis showed 5035 differentially expressed transcripts (Table S3A). The hierarchical cluster analysis of DEG showed two distinct clusters for 14 days of hESC differentiation representing up and down-regulated transcripts (Figure 2B). To further unravel the biological correlation of up-regulated genes, GO analysis were performed. BPs related to the neuron, skeletal system, vasculature and embryonic organ development were enriched among the up-regulated genes after 14 days of hESC differentiation (Table S4A). The neuron development BP gene expression pattern is represented in volcano plot (Figure 2C). A reduction of pluripotency genes is crucial for embryonic development and the array results corroborates a down-regulation of POU5F1, NANOG, and LIN 28 (Figure 2D). For proteomic analysis, Delta2D analysis (p≤0.01) of 2DE gels (as mentioned in methods) for hESCs and 14-day old EBs identified 281 regulated spots. A total of 61 protein spots were selected for further mass spectrometry. The spots encompassed 33 up-regulated and 28 down-regulated proteins, at a fold change ≥±1.5 (Table S5). The comparative analysis of genomics and proteomics for hESC differentiation unveiled 10 commonly regulated genes (Table 1). These included PAK2 and ENO2, which are highly expressed during neuronal development [29], [30]. Among the 33 up-regulated proteins, 11 were shown to be involved in neuronal differentiation and brain development, particularly the Reelin pathway regulators PAFAH1B2 and PAFAH1B3 (Figure 2E). Within down-regulated proteins, POU5F1 regulators (such as TPIM28, PKM2 and RBM14) have been identified in 14-day-old EBs, suggesting a progressive differentiation of the hESCs (Figure 2F).

**Table 1:** The common differentially regulated gene (mRNA (n = 3), (p≤0.05) and protein (n = 3), (p≤0.01)) expression pattern after 14 days differentiation.

Protein Name	Gene symbol	Spot ID	Score	Mass	Matches	Sequences	emPAI	Protein description	Protein FC	mRNA FC	Regulation
**AINX_HUMAN**	**INA**	**22502**	**228**	**55357**	**20 (9)**	**20 (9)**	**0.68**	**Alpha-internexin OS = Homo sapiens GN = INA**	**3.82**	**4.5**	**up**
**CLIC4_HUMAN**	**CLIC4**	**23571**	**205**	**28754**	**10 (7)**	**8 (6)**	**1.15**	**Chloride intracellular channel protein 4 OS = Homo**	**−2.77**	**−4.9**	**down**
**ENOG_HUMAN**	**ENO2**	**22823**	**340**	**47239**	**23 (11)**	**16 (8)**	**0.96**	**Gamma-enolase OS = Homo sapiens GN = ENO2**	**2.45**	**2.0**	**up**
**DC1L2_HUMAN**	**DYNC1LI2**	**22671**	**156**	**54066**	**6 (5)**	**6 (5)**	**0.34**	**Cytoplasmic dynein 1 light intermediate chain**	**3.68**	**2.1**	**up**
**EZRI_HUMAN**	**EZR**	**22172**	**436**	**69370**	**41 (18)**	**32 (15)**	**1.1**	**Ezrin OS = Homo sapiens GN = EZR PE = 1 SV = 4**	**−9.49**	**−2.4**	**down**
**PAK2_HUMAN**	**PAK2**	**22485**	**166**	**58006**	**18 (12)**	**17 (11)**	**0.94**	**Serine/threonine-protein kinase PAK 2 OS = Homo**	**1.82**	**2.2**	**up**
**PRDX2_HUMAN**	**PRDX2**	**23794**	**485**	**21878**	**30 (18)**	**12 (10)**	**5.39**	**Peroxiredoxin-2 OS = Homo sapiens GN = PRDX2**	**2.12**	**2.1**	**up**
**SERC_HUMAN**	**C8orf62**	**23725**	**169**	**40397**	**9 (8)**	**8 (7)**	**0.88**	**Phosphoserine aminotransferase OS = Homo**	**−2.67**	**−2.7**	**down**
**TAGL3_HUMAN**	**TAGLN3**	**23782**	**176**	**22458**	**15 (9)**	**11 (8)**	**2.05**	**Transgelin-3 OS = Homo sapiens GN = TAGLN3**	**2.58**	**15.5**	**up**
**TKT_HUMAN**	**TKT**	**22360**	**461**	**67835**	**35 (24)**	**23 (17)**	**1.7**	**Transketolase OS = Homo sapiens GN = TKT PE**	**−25.0**	**−27.4**	**down**

### Dose-response gene expression profiling in response to thalidomide

**Figure 3:**
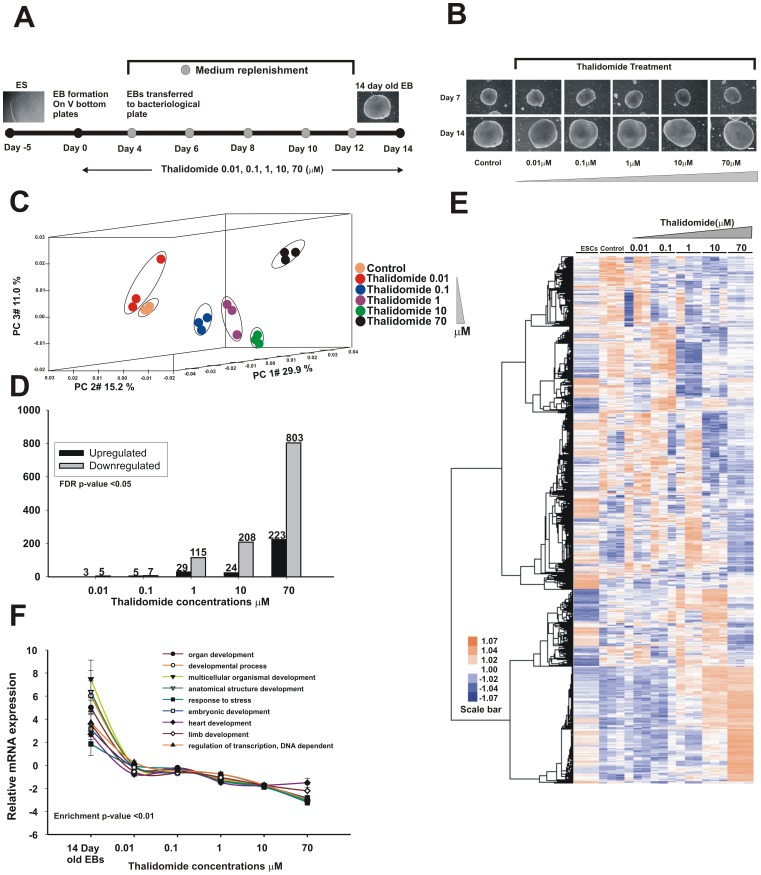
Dose-dependent gene expression response of thalidomide in hESC differentiation. **A**, The experimental approach of hESC differentiation and thalidomide treatment. **B**, Selected thalidomide concentrations do not induce any cytotoxicity in hESC differentiation. The scale bar represents 250 µm. **C**, 3D PCA showed that thalidomide treatment induces the changes in the gene expression pattern with increasing concentration. The 3 biological replicates for each sample group are showed in single colour. **D**, The number of DEG progressively increased with thalidomide concentration. Black shows up-regulated and grey shows down-regulated transcripts. Fold change ≥2 or ≤−2, (p≤0.05). **E**, An unsupervised hierarchical clustering of DEG demonstrated a dose-dependent repression. The highly expressed genes in untreated control were repressed for thalidomide in a concentration dependent manner. Data represents from 3 biological replicates. **F**, To quantify the BP the average relative fold change values were calculated manually (for all transcripts present in each GO) and represented for 14 day old EBs and thalidomide treatment. The error bar represents SEM from fold change values for transcripts belong to each GO.

Five concentrations were selected for the dose-response study and the differentiation scheme in presence of treatment was briefly demonstrated in Figure 3A. The morphology of EBs for all concentrations (0.01 µM, 0.1 µM, 1 µM, 10 µM and 70 µM) showed no reductions or alterations in the EB size due to cytotoxicity (Figure 3B). To determine the cytotoxic concentration, hESCs were treated with higher concentrations of thalidomide (up to 1 mM) and no cytotoxicity in Resazurin reduction assay was observed (data not shown).

To visualise the global gene expression pattern, principal component analysis (PCA) was performed. PCA projects the high content data with clustering of similar expression pattern of samples. Figure 3C shows merely any changes at the lowest concentration and at higher concentration significant difference from control was observed. The samples subjected to PCA demonstrate larger variances (PC#29.9%) at 1 µM and above concentrations. At a concentration of 0.01 µM, 5 DEG were observed and therefore was considered to be a low observed adverse effect level (LOAEL) concentration. The numbers of dysregulated transcripts increased progressively with thalidomide treatment and the highest thalidomide concentration (70 µM) yielded 803 down- and 223 up-regulated genes (Figure 3D) (Table S3A to S3F).

### Transcriptomic signatures and GO assessments for thalidomide

To determine the effect of thalidomide across different concentrations, F-statistics (From thalidomide dose response study) were applied (FDR corrected *p-*value≤0.05, 1-way ANOVA,) which resulted in 25433 DEG (Table S3G). The hierarchical clustering analysis of DEG showed dose dependent inhibition of gene signatures from 1 µM to 70 µM concentration (Figure 3E). To identify the functional relevance of these genes, functional annotation clustering analysis was performed separately for up- and down-regulated transcripts for 1, 10 and 70 µM (Table S6A to S6F). The representative GO for down-regulated genes are involved in heart development, limb development, vasculature development, skeletal system development, and DNA-dependent regulation of transcription (Table 2). The GO quantification of developmental biological processes showed that thalidomide induced significant developmental toxicity in a dose dependent manner (Figure 3F). The complete lists of GO are provided in Tables S6A to S6F for 1 to 70 µM.

**Table 2:** Selected down-regulated significant (p≤0.01) GO categories after thalidomide treatment.

Biological Process (GO)	Count			%			p-value			Representative Genes
	1 µM	10 µM	70 µM	1 µM	10 µM	70 µM	1 µM	10 µM	70 µM	
**heart development**	**8**	**18**	**-**	**7.84**	**10.65**	**-**	**5.31E-04**	**1.52E-10**	**-**	**RBP4, NRP1, TBX3, ERBB3, SOX6, ISL1, EDNRA, TDGF3, SALL4, HAND1, PKP2, GATA6, TDGF1, TGFBR3, PTCH1, ADAMTS1, PCSK5, MYH10, SMARCA4**
**limb development**		**9**	**7**	**-**	**5.32**	**1.25%**	**-**	**1.52E-05**	**0.003**	**SALL4, TBX3, HOXA10, HOXA9, LEF1, PTCH1, SP8, PCSK5, SMARCA4**
**vasculature development**	**11**	**12**	**-**	**10.78**	**7.1**	**-**	**5.68E-06**	**8.69E-05**	**-**	**NRP1, TBX3, EFNB2, APOB, HAND1, APOE, VEGFA, TGFBR3, FIGF, SMARCA4**
**skeletal system development**	**8**	**13**	**16**	**7.84**	**7.69**	**2.87%**	**0.005**	**1.76E-04**	**0.005**	**RBP4, TBX3, INS-IGF2, IGF2, SOX6, FRZB, COL5A2, HOXA10, HOXA9, PCSK5, IGFBP3, SPP1**
**developmental process**	**-**	**10**	**169**	**-**	**5.91**	**30.29%**	**-**	**0.002**	**7.85E-13**	**AFP, KRT18, FST, IGFBP3, HOXA7, GATA6, HOXC9, ANGPT1, HOXB9, HOXB8, NES, HES5, RAB23, HOXB6, RELN, SEMA6A, KRT19, TBX3**
**multicellular organismal process**	**-**	**9**	**159**	**-**	**5.32**	**28.49%**	**-**	**4.01E-04**	**1.08E-05**	**SERPINA1, HOXC6, SPARC, HOXD4, DICER1, POSTN, APOA4, LEF1, FABP7, FGA, HES5, RAB23, HOXB6, RELN, PITX2, SEMA6A,**
**embryonic development**	**-**	**19**	**25**	**-**	**11.24**	**4.48%**	**-**	**1.73E-08**	**2.26E-05**	**TDGF3, HAND1, TBX3, PCDH8, RAB23, ZEB1, HOXB6, THBD, AXIN2**
**regulation of transcription, DNA-dependent**	**-**	**17**	**96**	**-**	**10.05**	**17.20%**	**-**	**5.68E-05**	**0.009**	**SOX1, FST, LEF1, HOXC6, CREB1, HOXA9, HOXD4, HES5, RBBP4, HOXB6, TCF7L2, E2F7, HOXA7, HOXA5, GATA6, PSIP1, HOXC9, ZNF367, PITX2, BNC1, ZMYM2, RBL1, HOXB3**
**organ development**	**-**	**9**	**81**		**5.32**	**14.52%**	**-**	**0.002**	**1.13E-09**	**DCN, SRGN, HMGCR, COL6A3, KRAS, GREM1, SPP1, FST, AFP, SPARC, HES5, IGFBP3, SEMA6A, KRT19, PTCH1, FRZB, BMPR1B**

GO categories were identified with DAVID analysis using DEG from the respective treatment concentration.

### Transcription factor analysis

GO analysis of the down-regulated transcripts (at 70 µM) uncovered 96 transcripts for DNA-dependent, regulation of transcription (Table 2). An independent functional annotation analysis shows that these factors are involved in embryonic organ development, skeletal system development, sensory organ development and pattern specification processes (Table S7). The list of genes contributing to each GO consists of Homeobox, Tbox, helix loop helix and members of the zinc finger transcriptional factor families.

### Perturbation of heart, limb development and WNT related genes

**Figure 4:**
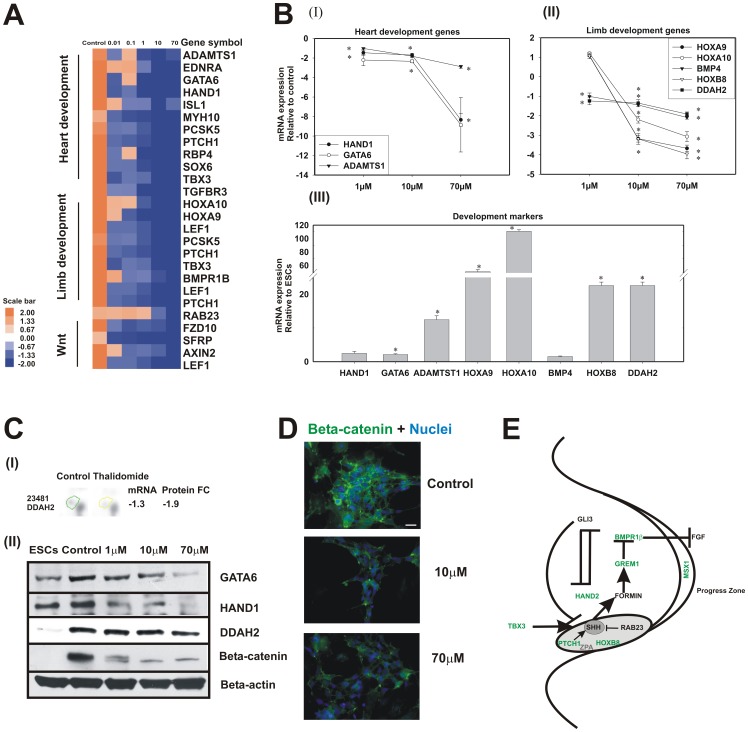
Thalidomide perturbed heart, limb development and WNT signalling associated genes. **A**, The heat map shows the microarray expression pattern for the selected genes for heart, limb development (from Table S6D, S6F) and WNT signalling (from Table S9). Since multiple genes are involved in various developmental processes few redundant genes are present. The up-regulated genes in control were repressed in a dose dependent manner. **B**, The representative heart (I), limb development (II) associated genes were analysed using RT-qPCR analysis with an independent experiment. Thalidomide responsive genes were shown in I and II (*p-value≤0.01, thalidomide-treated vs untreated 14-days old EBs) and 14 days old EBs gene expression were shown in (III) *p-value≤0.01, 14 days old EBs vs ESCs . The error bars represents the SEM from 3 technical replicates. **C**, The mass spectrometry results showed down-regulation of DDAH2 gene (n = 3), (p≤0.01). Figure C (I) represents spot analysis of DDAH2, mRNA and protein fold change (FC) values. C (II) The immunoblotting analysis of representative genes for thalidomide treatment. **D**, The immunoflourescence results shows the localisation of β-catenin and the gradient suppression at 70 µM thalidomide treatment. The scale bar represents 20 µm. **E**, The figure representing limb bud patterning was adapted from [40] and modified. The figure shows the molecular markers that SHH and ZPA (essential for limb development) were affected by thalidomide treatments. The green colour coded genes are more than −1.8 fold down-regulated.

The differential expression in heart and limb development BP coincides with clinical evidence of thalidomide toxicity [9], [10]. The representative genes from the respective BP uncovered transcriptional factors (such as GATA6, HAND1 for heart development and, HOXA10, HOXA9, TBX3, and HOXB8 for limb development) which were up-regulated in untreated 14-day differentiated cells. In contrast, negative regulation of these genes was observed for thalidomide treatment (Figure 4A). Additionally, we found that groups of genes involved in WNT signalling pathway, such as FZD10 (a WNT activator), AXIN (a β-catenin assembly stabiliser) and TCF/LEF (a WNT signalling target) were down-regulated by 2-fold (Figure 4A). The Delta 2D analysis of 2DE gels for thalidomide (10 µM) revealed 6 down-regulated protein spots at p≤0.01 and fold change ≥±1.3 (Table S8). These spots were selected for mass spectrometry analysis (Figure S3). The results showed a 1.9-fold down-regulation of dimethylarginine dimethylaminohydrolase 2 (DDAH2), which regulates the nitric oxide synthase via destruction of asymmetric dimethylarginines and is involved in limb development (Figure 4B (II)). Regulation of selected markers for heart and limb development was confirmed with immunoblotting and RT-qPCR analysis (Figure 4B, 4C). To find the interference of thalidomide in early cardiac development an independent temporal and dose response experiment was performed. The RT-qPCR analysis demonstrates cardiac specific transcription factors such as NKX2.5, GATA4, GATA6 and HAND1 were down-regulated (Figure S4). The cellular localization of β-catenin is showed with immunostaining (Figure 4D). The dysregulated genes related to anteroposterior (A–P) limb development in response to thalidomide are represented in Figure 4E.

### Thalidomide-induced dysregulation of glutathione transferases (GST) and nucleocytoplasmic trafficking

**Figure 5:**
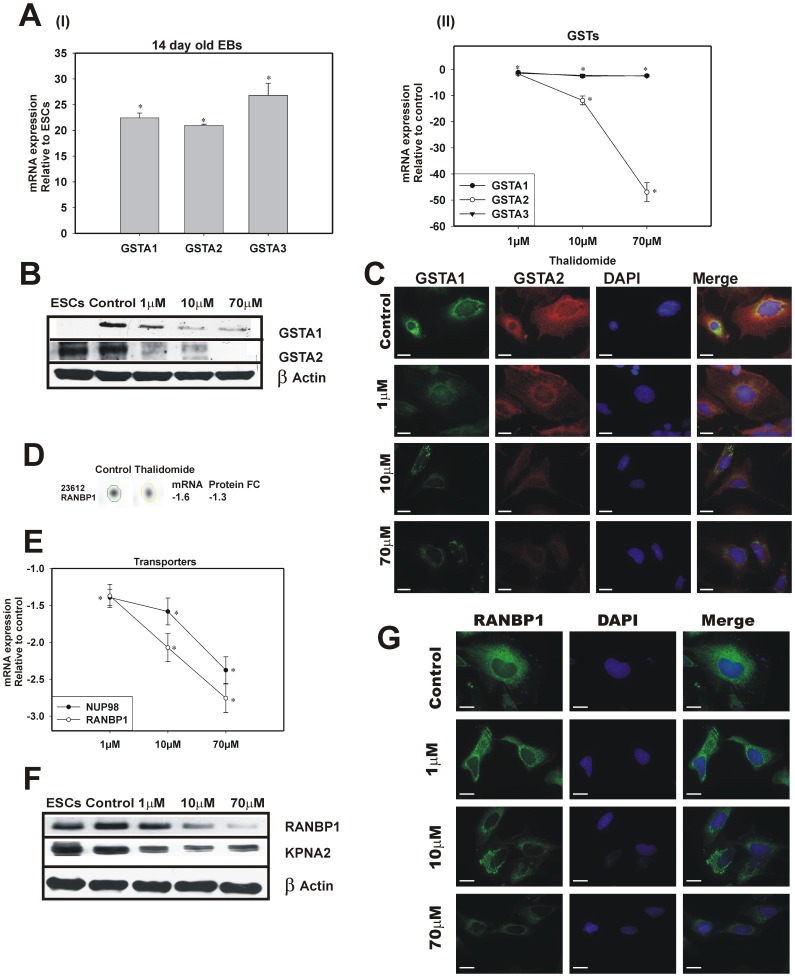
Thalidomide suppressed the GST genes and nucleocytoplasmic transporters at mRNA and protein level. To validate microarray expression. **A**, (I) (RT-qPCR analysis) 14 day's hESC differentiation showed higher GST expression (*p-value≤0.01, 14 days old EBs vs ESCs) and (II) thalidomide treatment (**p-value≤0.05, thalidomide-treated vs untreated 14-days old EBs) repressed GST genes more than −2 fold change. The error bars represent the SEM from 3 technical replicates from an independent experiment. **B**, Immunoblotting analysis of GSTA1/2 confirms thalidomide induced perturbation at protein concentration. **C**, The cellular localisation of GSTA1/2 showed pronounced concentration of GSTA on nuclear membrane in control is attenuated for 10 and 70 µM. The scale bar represents 20 µm. **D**, The genomic and mass spectrometry analysis showed down-regulation of RANBP1. **E**, Investigation of transporters genes for thalidomide treatment with RT-qPCR analysis (from independent experiment). The error bars represent the SEM from 3 technical replicates. **F**, Thalidomide mediated protein concentration changes were shown with immunoblotting analysis. **G**, The sub cellular localisation of RANBP1 is demonstrated with immunoflourescence analysis. The cytoplasmic enrichment of RANBP1 gradient is repressed with thalidomide treatment. The scale bar represents 20 µm.

Glutathion depletion or oxidation and ROS generation is another proven mechanism for thalidomide teratogenecity [20], [31]. From microarray experiment we found GSTA1 was over expressed by 3 fold in 14 days old EBs and down-regulated by 2-fold change for thalidomide. The GST family are involved in detoxifying xenobiotics and inactivating secondary metabolites (such as hydroperoxides and epoxides) during oxidative stress [32]. To further reconfirm glutathione family genes expression such as GSTA1, GSTA2 and GSTA3 real time PCR and immunoblotting analysis were performed which confirm dose dependent repression for thalidomide (Figure 5A, 5B). To find the cellular localisation of GSTA1 and GSTA2, immunoflourescence analysis was performed for control which shows an enrichment of these respective proteins in nuclear membrane. In response to thalidomide positive signals were found in the nuclear membrane; however the enrichment of proteins collapsed (Figure 5C).

The perturbation of generation and clearance of ROS in cellular machinery leads to the pronounced effect of oxidative stress. But the well known fact of oxidative stress induced nucleocytoplasmic traffic dysregulation was not studied previously in response to thalidomide. Microarray analysis showed repression of trafficking genes KPNA2, RANBP1, NUP98 and NUP160 by more than −1.5 fold (Table S3G). A mass spectrometry analysis showed the down-regulation of RANBP1 (Figure 5D). A RAN binding protein present in the cytoplasm which is required for carriers nucleoporins and adaptor importin-α (KPNA2) [33]. The RT-qPCR and immunoblotting analysis confirmed the concentration dependent down-regulation of trafficking genes (Figure 5D, 5E, 5F). Immunoflourescence analysis demonstrated that thalidomide induced alteration of cytoplasmic RANBP1 gradient (Figure 5G).

## Discussion

The present time kinetic experiment demonstrates that the optimum differentiation time point, which is relevant for developmental toxicity approaches, was reached after day 12. Additionally, the expression pattern was consistent up to day 21. The up-regulated transcripts of 14-day differentiated cells belong predominantly to various embryonic developmental biological processes, such as skeletal, neuronal, and respiratory system development. The significant number of transcripts expressed during differentiation emphasises a critical window to monitor molecular mechanisms and developmental pathways induced by developmental toxicants. Our comparative 2DE proteomics analysis resulted in 33 up-regulated proteins. Eleven of these represented proteins are involved in neuronal development (Figure 2E). Among the 11 proteins, we found 2 proteins that belong to the Reelin pathway (PAFAH1B2 and PAFAH1B3) and that regulate brain development. PAK4 was also identified, which targets Rho GTPases, such as CDC42, that are required for neuronal development and axon outgrowth [29], [34]. Up-regulation of RELN and CDC42 at the protein level also confirmed with mRNA levels uncovered by the microarray data (Figure 2C).

To reveal the dose dependent gene expression pattern during hESC differentiation 5 concentrations which represent clinical plasma concentrations for therapeutic purposes were selected. As previously described, the mean steady state concentration for lower and higher doses of thalidomide ranges between 1 to 10 µM [35], [36]. Highly expressed developmental genes at 14 days were down-regulated after treatment with increasing concentrations of thalidomide. Notably, BP and GO quantification results showed that thalidomide affected embryonic, heart and limb and development (Table 2, Figure 3F). Dose-response genomic data has an advantage because it demonstrates the specific dose(s) affecting the corresponding BP. The present study shows the alterations in BP associated with mammary glands at a dose of 0.88 µM (Table S9 BMD data), which was observed previously in the beagle dog [12]. Perturbation of heart and limb development was consistently shown for thalidomide both *in vivo* and *in vitro* [17], [37]–[39]. Therefore, these findings obtained under *in vitro* developmental conditions using hESCs suitably recapitulate the disturbances in heart and limb development observed in *in vivo* animal studies [11], [15], [39].

Canonical embryonic limb bud development in vertebrates proceeds along the proximal to distal (P–D), A–P and dorsal to ventral (D–V) axes [40]. P–D limb development is regulated by apical ectodermal ridge (AER) and FGF signalling [40]. The A–P limb development depends on the expression of SHH and zone of polarising activity (ZPA). Using 14-day EBs, we observed high expression levels of the transcription factors HOXB8 and HAND2, which are essential for ZPA and SHH expression (a regulator of TBX3 and PTCH1) [41], [42]. GREM1, a negative regulator of BMP, was downregulated by 10-fold. The similar expression pattern of 14-day EBs and A-P limb development genes *in vivo* suggest that this model is suitable to predict specific effects in A–P limb development. These genes were significantly dysregulated by thalidomide in a dose-dependent manner (Figure 4E). Cereblon (CRBN) forms an E3 ubiquitin ligase complex with CUL4A and DNA binding protein1 and was identified as one of the molecular targets for thalidomide. It is also expressed during limb outgrowth in zebrafish and chicks, which depends on FGF signalling [43]. Because our model does not express genes involved in P–D limb development, such as FGF8 or CRBN, this mechanism of developmental toxicity cannot be confirmed. External addition of growth factors may enable our model to encompass P–D patterning.

Interestingly, expression of the homeobox transcription factors HOXA, HOXD and HOXB8 is also affected by thalidomide treatment in differentiated hESCs (Table S7, Figure 4C). It is well known that HOXA, HOXD and HOXB8 promote limb morphogenesis (reviewed in [44]) and early limb-bud formation [44], [45]. WNT signalling controls the dynamic limb development processes such as initiation, patterning and differentiation [46]. The attenuation of markers involved in WNT signalling was observed in the present study (Figure 4A, 4C, 4D) which coincides with the previous *in vitro* studies [17], [18]. DDAH1/2 catalyses the hydrolysis of asymmetric dimethylarginine (an endogenous inhibitor of the nitric oxide synthase [47]) and is expressed in limb-bud patterning. We observed perturbations of DDAH2 at both the gene and protein level (Figure 4B, 4C). Perturbations of A-P limb bud development genes in thalidomide-treated hESCs are shown in Figure 4E.

Transcription factor HAND1 expresses highly in developing left ventricle and shows profound expression in the right ventricle. The chick embryo studies showed knock down of HAND1 attenuates heart development [48]. GATA6 transcription factor expresses in the early mesoderm development during the heart specification and is reduced during the terminal differentiation [49]. The higher expression of these transcription factors in 14 days differentiation showed the specification of early embryonic development. A further differential expression with thalidomide demonstrates the causes of defects in early cardiac development which was consistent in an *in vivo* studies [15]. MSX1 plays an important role in cleft palate and craniofacial development, as demonstrated by MSX1-deficient mice [50]. The high gene expression levels of MSX1 in differentiated hESCs were repressed in the presence of thalidomide. These findings correlate well with cleft palate abnormalities observed in children's exposed to thalidomide [51], [52].

Many molecular mechanisms have been proposed to explain the teratogenic effects of thalidomide including free radical oxygen species (ROS)-mediated oxidative DNA damage, oxidative stress-mediated GSH depletion and NF-kB attenuation [16], [20], [21]. Most of the previous studies [21] mainly focus on the GSH depletion in response to thalidomide via ROS generation. Interestingly, in the present study we found reduced expression of GSTA (Figure 5A, 5B, 5C) for thalidomide which is required for secondary cellular oxidative stress [32]. Oxidative stress affects basic cellular physiology, such as nuclear and cytoplasmic trafficking [33]. Any type of perturbation in the nuclear traffic machinery, a hallmark of oxidative stress, limits the efficiency of transport, which controls development, differentiation and transformation [53].

The down-regulation of RANBP1 (a RAN GTPase binding protein) (Figure 5D) in 2DE analysis and gene expression pattern of importin-α (KPNA2) and nucleoporins (NUP98) was confirmed with real time and western blot analysis (Figure 5E, 5F). Importin-β and the adaptor importin-α are crucial components of the nuclear transport apparatus. Importins interact exclusively with the GTP bound form of the Ran GTPase. This process enables exportation of the importins from the nucleus in association with Ran-GTP, thereby regulating nucleocytoplasmic transport of cytosolic proteins through the nuclear pores [30]. A gradient of high Ran-GTP in the nucleus, versus high Ran-GDP in the cytoplasm, is established by cytoplasmic localisation of Ran-binding protein 1 (RanBP1) [30]. This results in a release of Ran-GTP from importins. It is well known that interference in the nucleocytoplasmic trafficking pathway affects embryonic development and differentiation [53], and it partially explains the teratogenic effects of thalidomide.

A recent publication [54] demonstrated that spontaneous differentiation of hESCs in the presence of toxicants dysregulated genes related to specific target tissues. Although this method can demonstrate the toxic effect of drugs for preliminary screening, a 7-day differentiation of the hESCs and a single-dose treatment assay limited the scope of the study. This study showed neither a critical dose for toxicity nor molecular mechanisms related to toxicants, including thalidomide. Differentiation of ESCs is a dynamic process, implicating the differential expression of thousand of genes in a very hierarchical manner. Therefore, missing the optimal time point or the appropriate concentration of the toxicant is critical for establishing a consistent and robust *in vitro* ESC-based toxicological model. Our independent time kinetic microarray study of hESC differentiation demonstrated that expression of embryonic development-related genes increased exponentially up to day 12. Following day 12, the global expression pattern was sustained for the remaining days (up to day 21) in hierarchical clustering (Figure 1C). In previous study, we demonstrated that a 7-day differentiation of hESCs is not sufficient to monitor the developmental adverse effects of cytosine arabinoside [24]. In summary, this *in vitro* study demonstrated that the perturbation of limb and heart formation, the nucleocytoplasmic trafficking and GST expression (with respective markers at the genomic and proteomic level) may represent a critical embryotoxicity for thalidomide. One of the main advantages of this alternative approach is the application of human ESCs. Human ESCs better represent human cellular physiology, can improve the safety of drug testing and can partially explain the mechanism(s) of a specific toxicant.

## Supporting Information

Figure S1 **The time kinetic experiment showed progressive expression of development related genes.** To find the common genes among different days of differentiation intersect analysis was performed. We found regulated transcripts (n = 3), (p≤0.05) constantly expresses after day 12.
(TIF)

Figure S2 **A typical 2DE image of undifferentiated hESC (I) and 14-day-old EBs (II) are represented.** The down (I) and up-regulated (II) spots are denoted in the gel pictures. IP-isoelectric pH value. MW-molecular weight. The differentially regulated proteins (n = 3), (p≤0.01) are from 3 biological replicates.
(TIF)

Figure S3 **A typical 2DE image of control (I), thalidomide treatment (II) and percentage volume (III) for differentially regulated spots (n = 3), (p≤0.01).** The error bars represents SEM from 3 independent biological replicates.
(TIF)

Figure S4 **Perturbation of thalidomide in early cardiac development.** For time and dose response experiment, representative cardiac specific transcription factors were analysed with RT-qPCR Bar represents mean value from an independent experiment of 3 technical replicates (*p-value ≤0.01, thalidomide-treated vs untreated 14-days old EBs) and error bar shows SEM. Y-axis represents relative mRNA expression compared to control. X-axis shows temporal analysis for thalidomide treatment.
(TIF)

Table S1 **Primer sequences used for real time PCR analysis.**
(DOC)

Table S2 **A) The F-statistics output for the time kinetic experiments. B to I)**. The T-statistics output for the time kinetic experiments.
(XLS)

Table S3 **A to F) The T-statistics output for dose-response analysis.** G) The F-statistics output for the dose-response analysis.
(XLS)

Table S4 **The GO analysis for the hESC differentiation over 14 days.** The DEG obtained from Table S3A. Table 4A) The up-regulated genes. 4B) The down-regulated genes.
(XLS)

Table S5 **The 61 regulated protein spots identified from 2DE analysis of hESC differentiation over 14 days using mass spectrometry.**
(XLS)

Table S6 **GO analysis for thalidomide treatment (1, 10 and 70 µM).** The DEG obtained from Table S3F. 6A) 1 µM up-regulated genes. 6B) 1 µM down-regulated genes. 6C) 10 µM up-regulated genes. 6D) 10 µM down-regulated genes. 6E) 70 µM up-regulated genes. 6F) 70 µM down-regulated genes.
(XLS)

Table S7 **Selected GO categories for 96 DNA dependent transcription factors after 70 µM treatment.** Thalidomide treatment results in the down-regulation of 96 DNA-dependent transcriptional factors (from table 2). Additional GO analysis demonstrated that these transcriptional factors are related to various embryonic developmental processes.
(DOC)

Table S8 **The 6 regulated protein spots, identified from 2DE using mass spectrometry, for thalidomide treatment.**
(XLS)

Table S9 **The BMD assessment as described below for GOs (biological processes and cellular component) related to embryonic development and the corresponding BMD mean and lower confidence BMD mean.**
(DOC)
