# Supplementary figures and images for: Identification of Thalidomide-Specific Transcriptomics and Proteomics Signatures during Differentiation of Human Embryonic Stem Cells
Source: PLoS One. 2012 Aug 28;7(8):e44228. doi: 10.1371/journal.pone.0044228 (PMC3429450; doi:10.1371/journal.pone.0044228)

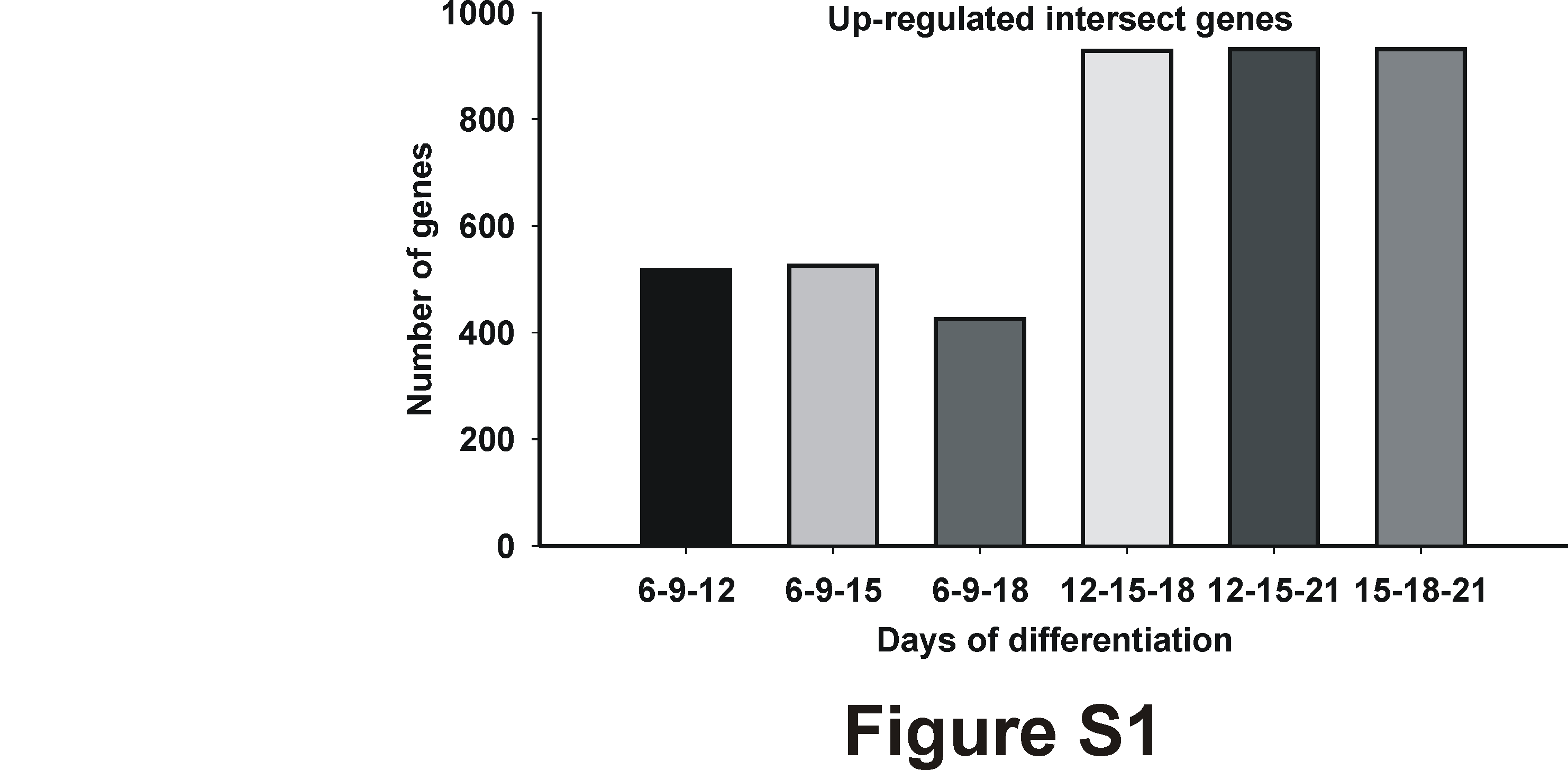

Supplement: Figure S1 — The time kinetic experiment showed progressive expression of development related genes. To find the common genes among different days of differentiation intersect analysis was performed. We found regulated transcripts (n = 3), (p≤0.05) constantly expresses after day 12. (TIF) [file pone.0044228.s001.tif]

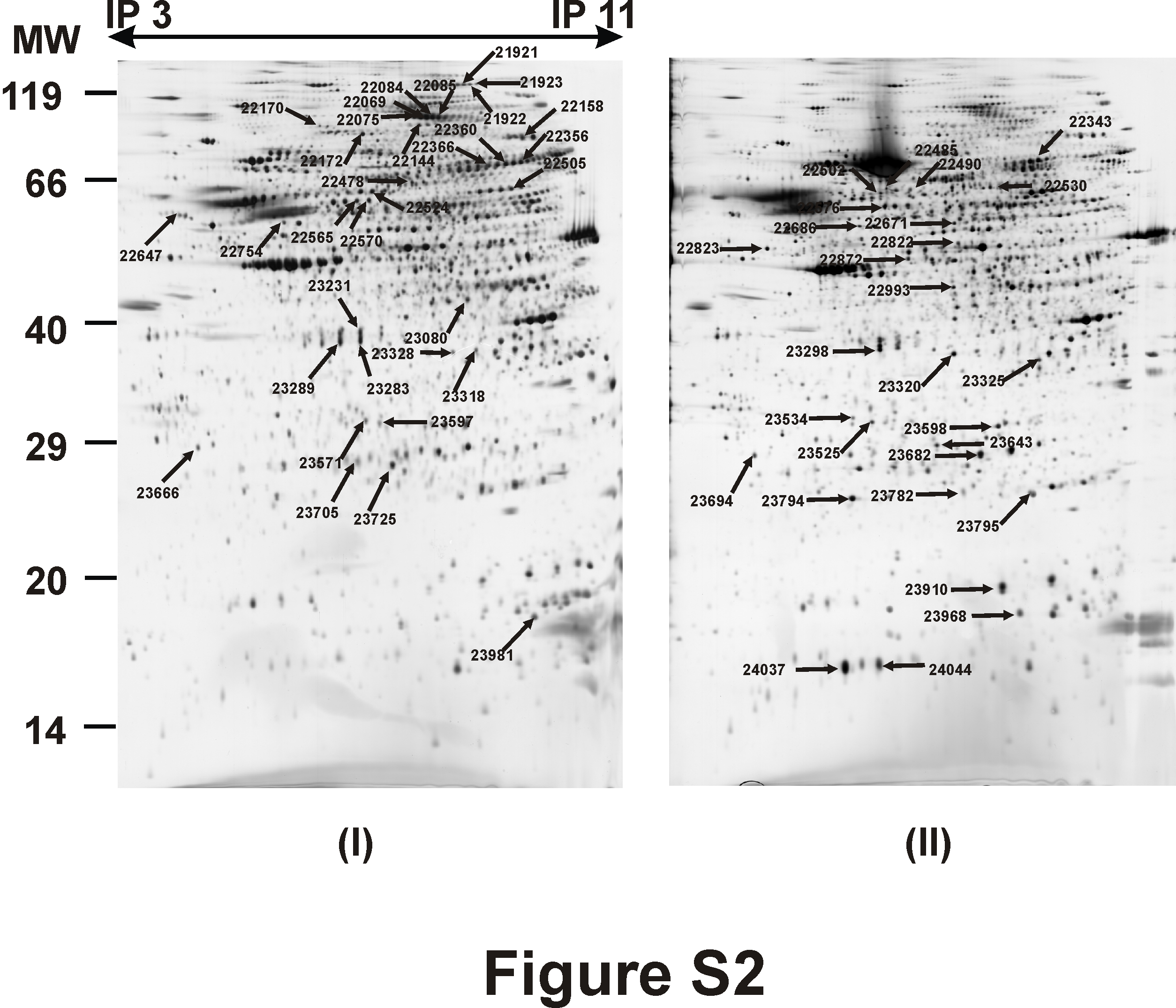

Supplement: Figure S2 — A typical 2DE image of undifferentiated hESC (I) and 14-day-old EBs (II) are represented. The down (I) and up-regulated (II) spots are denoted in the gel pictures. IP-isoelectric pH value. MW-molecular weight. The differentially regulated proteins (n = 3), (p≤0.01) are from 3 biological replicates. (TIF) [file pone.0044228.s002.tif]

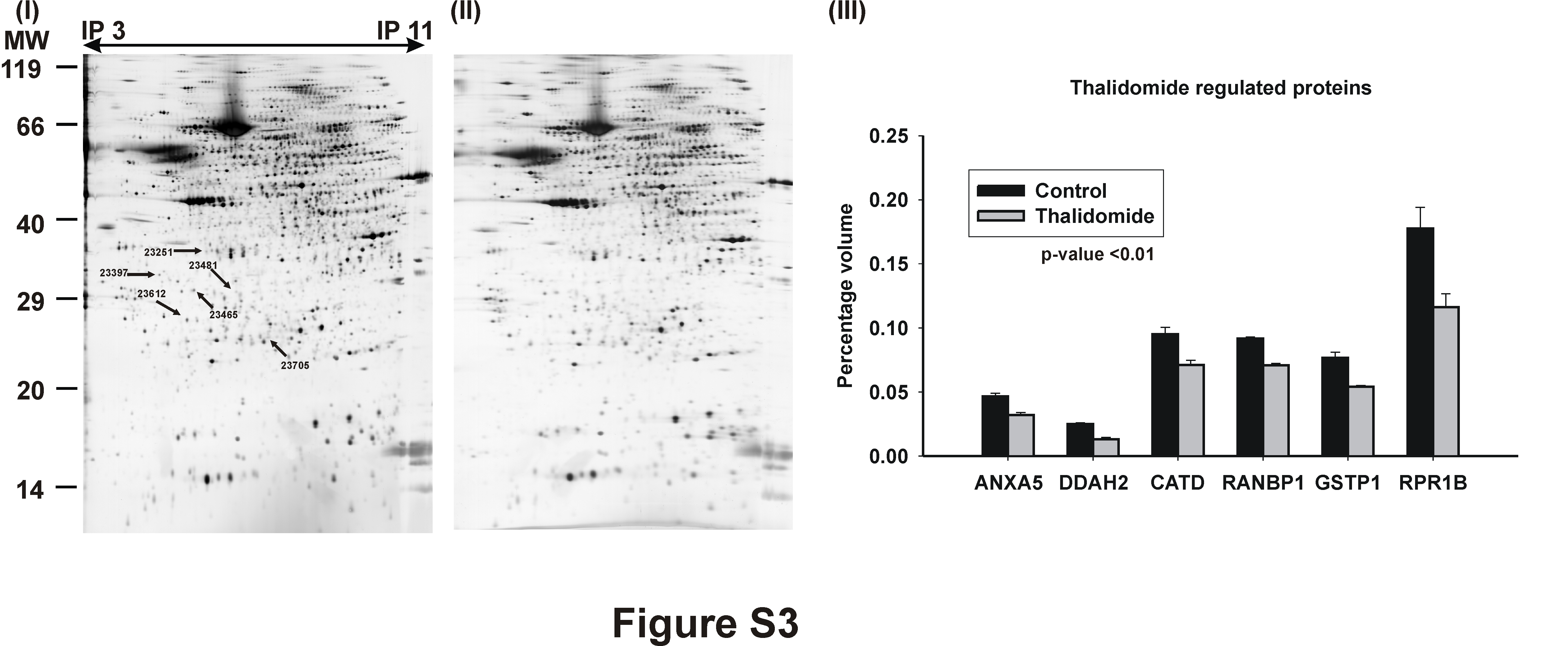

Supplement: Figure S3 — A typical 2DE image of control (I), thalidomide treatment (II) and percentage volume (III) for differentially regulated spots (n = 3), (p≤0.01). The error bars represents SEM from 3 independent biological replicates. (TIF) [file pone.0044228.s003.tif]

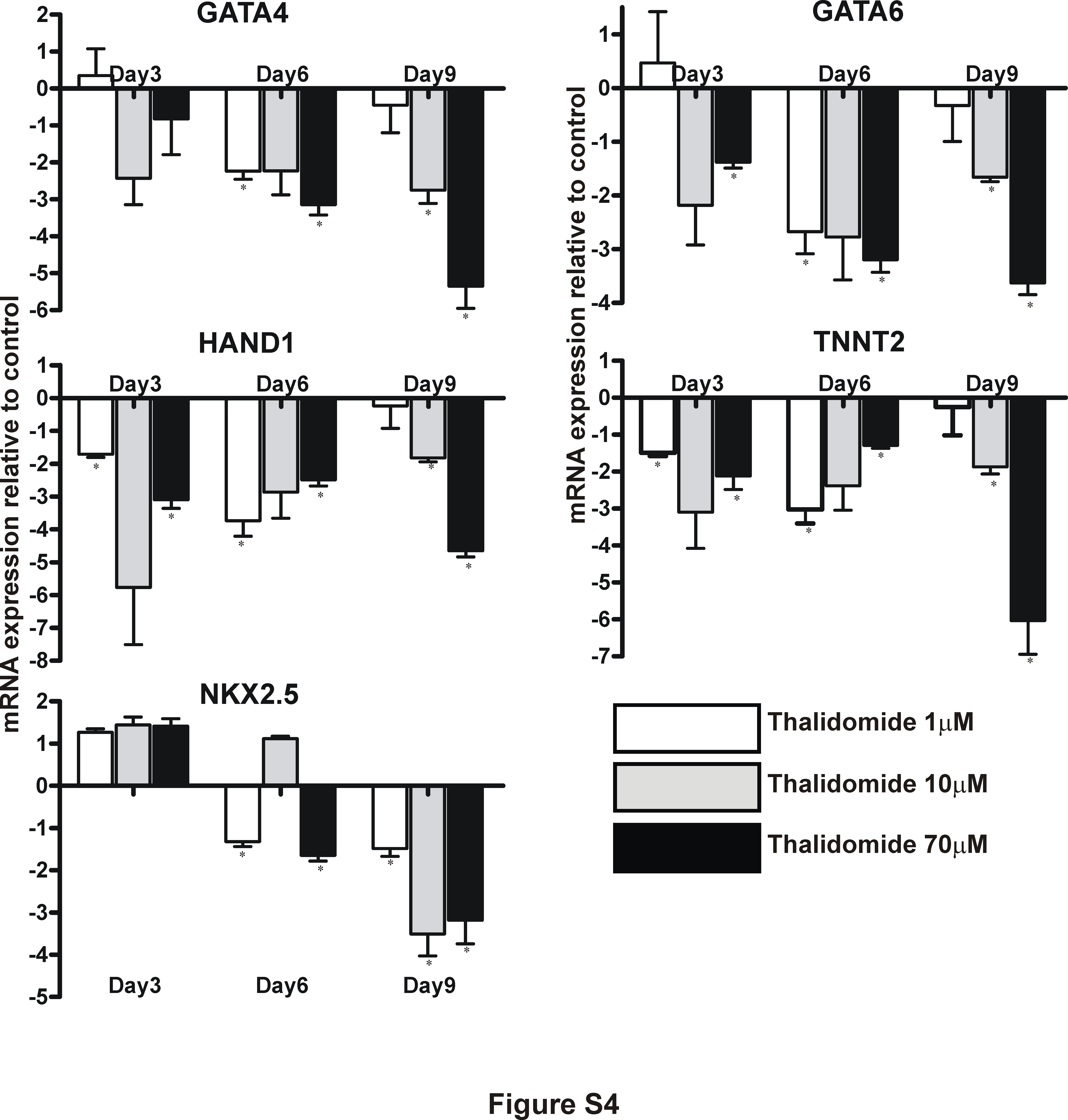

Supplement: Figure S4 — Perturbation of thalidomide in early cardiac development. For time and dose response experiment, representative cardiac specific transcription factors were analysed with RT-qPCR Bar represents mean value from an independent experiment of 3 technical replicates (*p-value ≤0.01, thalidomide-treated vs untreated 14-days old EBs) and error bar shows SEM. Y-axis represents relative mRNA expression compared to control. X-axis shows temporal analysis for thalidomide treatment. (TIF) [file pone.0044228.s004.tif]
