# Supplementary material for: Identification of Thalidomide-Specific Transcriptomics and Proteomics Signatures during Differentiation of Human Embryonic Stem Cells
Source: PLoS One. 2012 Aug 28;7(8):e44228. doi: 10.1371/journal.pone.0044228 (PMC3429450; doi:10.1371/journal.pone.0044228)
Supplement: Table S1 — Primer sequences used for real time PCR analysis. (DOC) [file pone.0044228.s005.doc]

| **Gene Symbol** | **Forward Sequences** | **Reverse Sequences** |
| --- | --- | --- |
| GATA6 | GCAAAAATACTTCCCCCACA | GCACGGAGGACGTGACTT |
| HAND1 | CCAAGGATGCACAGTCTGG | CGGTGCGTCCTTTAATCCT |
| ADAMTS1 | GGGGGAAATGGATCTACTTGT | GCAAGAAAGCTGCCATTGTT |
| HOXA9 | AGAATGAGAGCGGCGGAGACAA | CTCTTTCTCCAGTTCCAGGGTC |
| HOXA10 | CTTCCGAGAGCAGCAAAGCCTC | TCCAGTGTCTGGTGCTTCGTGT |
| BMP4 | CTGGTCTTGAGTATCCTGAGCG | TCACCTCGTTCTCAGGGATGCT |
| HOXB8 | GTCGCCCACACAGCTCTTCCC | AATAGGAACTCCTTCTCCAGCTC |
| DDAH2 | CTTTCTTCGTCCTGGGTTGCCT | CTCCAGTTCTGAGCAGGACACA |
| GSTA1 | GCAGACCAGAGCCATTCTCAAC | ACATACGGGCAGAAGGAGGATC |
| GSTA2 | CTGCCCTTTAGTCAACCTGAGG | ACAAGGTAGTCTTGTCCGTGGC |
| GSTA3 | GTCGCTATTTCCCTGCCTTCGA | GTTCCACCAGGCTAATGTCAGC |
| RANBP1 | ACCATGACCCTCAGTTTGAGCC | AGTGCCTCGCTCCTTCCATTCT |
| NUP98 | GGAACCTGTGTCTGCCTCAACA | CTTTGGAAGGCAGGCGACTGAA |
| GATA4 | GAGATGCGTCCCATCAAGAC | GGGAGACGCATAGCCTTGT |
| TNNT2 | AGTGGGAAGAGGCAGACTGA | CGAACTTCTCTGCCTCCAAG |
| NKX2.5 | TGCCTCTCCTTCTGAACCTTGG | GCGAAATCTGCCACCAGTTG |
| β-ACTIN | GCAAAGACCTGTACGCCAAC | ACATCTGCTGGAAGGTGGAC |

**Table S1**. Primer sequences used for real time PCR analysis
