# Supplementary material for: Identification of Thalidomide-Specific Transcriptomics and Proteomics Signatures during Differentiation of Human Embryonic Stem Cells
Source: PLoS One. 2012 Aug 28;7(8):e44228. doi: 10.1371/journal.pone.0044228 (PMC3429450; doi:10.1371/journal.pone.0044228)
Supplement: Table S7 — Selected GO categories for 96 DNA dependent transcription factors after 70 µM treatment. Thalidomide treatment results in the down-regulation of 96 DNA-dependent transcriptional factors (from table 2). Additional GO analysis demonstrated that these transcriptional factors are related to various embryonic developmental processes. (DOC) [file pone.0044228.s011.doc]

**Table S7. Selected GO categories for 96 DNA dependent transcription factors after 70 μM treatment. Thalidomide treatment results in the down-regulation of 96 DNA-dependent transcriptional factors (from table 2). Additional GO analysis demonstrated that these transcriptional factors are related to various embryonic developmental processes**

| **Term** | **Count** | **%** | **P-Value** | **Genes** |
| --- | --- | --- | --- | --- |
| anterior/posterior pattern formation | 16 | 17 | 2.60E-14 | TSHZ1, MLL, TBX3, LEF1, HOXB3, HOXC6, HOXC9, HOXB7, HOXA5, HOXB8, HOXD4, HOXA7, HOXB6, HOXA10, HOXA9, HOXB9 |
| embryonic organ development | 16 | 17 | 5.55E-13 | TSHZ1, MLL, EPAS1, ZEB1, TCF7L2, HOXB3, HOXC9, HAND1, HOXB7, HOXA5, HOXB8, HOXD4, HOXA7, HOXB6, ZFPM2, MYC |
| skeletal system development | 17 | 18.08 | 3.92E-10 | TBX3, SMAD5, SOX6, ZEB1, HOXC6, HOXB3, HOXC9, HOXB7, HOXA5, HOXB8, HOXD4, HOXA7, HOXB6, HOXA10, HOXA9, HOXB9, MYC |
| lung development | 6 | 6.38 | 5.64E-04 | TIMELESS, EPAS1, HOXA5, GATA6, ZFPM2, NFIB |
| sensory organ development | 8 | 8.5 | 9.40E-04 | TSHZ1, SOX1, HES5, BCL11B, MITF, TGIF2, ZEB1, MYC |
| gland development | 7 | 7.44 | 3.12E-04 | HOXB3, TBX3, HOXA5, CREB1, HOXA9, LEF1, HOXB9 |
| tube development | 8 | 8.5 | 7.43E-04 | TIMELESS, HAND1, TBX3, EPAS1, HOXA5, GATA6, ZFPM2, NFIB |
| in utero embryonic development | 6 | 6.3 | 0.00696431 | HAND1, TBX3, EPAS1, GATA6, ZFPM2, RBBP8 |
