# Supplementary material for: Identification of Thalidomide-Specific Transcriptomics and Proteomics Signatures during Differentiation of Human Embryonic Stem Cells
Source: PLoS One. 2012 Aug 28;7(8):e44228. doi: 10.1371/journal.pone.0044228 (PMC3429450; doi:10.1371/journal.pone.0044228)
Supplement: Table S9 — The BMD assessment as described below for GOs (biological processes and cellular component) related to embryonic development and the corresponding BMD mean and lower confidence BMD mean. (DOC) [file pone.0044228.s013.doc]

**Table S9**. The BMD assessment as described below for GOs (biological processes and cellular component) related to embryonic development and the corresponding BMD mean and lower confidence BMD mean

| **GO Term Name** | **All Genes** | **Number of genes** | **%** | **BMD Mean**  **μM** | **BMD SD**  **μM** | **BMDL Mean**  **μM** | **BMDL SD**  **μM** |
| --- | --- | --- | --- | --- | --- | --- | --- |
| mammary gland development | 54 | 10 | 18.52 | 0.88 | 1.19 | 0.61 | 0.84 |
| limb development | 93 | 10 | 10.75 | 3.04 | 4 | 2.07 | 2.82 |
| heart development | 202 | 19 | 9.41 | 6.13 | 12.62 | 4.14 | 8.36 |
| urogenital system development | 109 | 11 | 10.09 | 6.19 | 10.92 | 4.20 | 7.32 |
| skeletal system development | 347 | 39 | 11.24 | 7.31 | 17 | 4.29 | 8.59 |
| embryonic development | 528 | 61 | 11.55 | 7.74 | 9.92 | 5.22 | 6.43 |
| central nervous system development | 385 | 29 | 7.53 | 8.31 | 8.09 | 5.81 | 5.69 |
| reproductive structure development | 118 | 12 | 10.17 | 8.39 | 11.92 | 5.84 | 7.70 |
| Wnt receptor signalling pathway | 157 | 10 | 6.37 | 8.74 | 13.45 | 5.81 | 8.70 |
| organ development | 1724 | 150 | 8.70 | 8.75 | 13.37 | 5.92 | 8.18 |
| circulatory system process | 223 | 13 | 5.83 | 8.82 | 11.70 | 6.22 | 8.04 |
| ectoderm development | 183 | 15 | 8.20 | 10.65 | 14.90 | 7.21 | 9.60 |
| DNA repair | 287 | 24 | 8.36 | 10.74 | 7.98 | 7.69 | 5.58 |
| vasculature development | 283 | 30 | 10.60 | 10.99 | 13.63 | 7.60 | 9.16 |
| sensory organ development | 211 | 15 | 7.11 | 12.40 | 12.19 | 8.66 | 8.10 |
| cellular response to DNA damage stimulus | 335 | 26 | 7.76 | 12.62 | 10.40 | 8.90 | 6.96 |
| bone development | 164 | 15 | 9.15 | 13.65 | 25.83 | 7.63 | 12.70 |
| neuron projection | 324 | 28 | 8.64 | 14.13 | 22.46 | 8.78 | 11.48 |
| cell junction | 500 | 40 | 8.00 | 12.29 | 17.93 | 8.15 | 9.68 |
| cell surface | 304 | 20 | 6.58 | 6.99 | 7.20 | 4.63 | 4.41 |
| collagen | 35 | 10 | 28.57 | 12.76 | 16.91 | 8.61 | 11.12 |
| dendrite | 152 | 10 | 6.58 | 9.67 | 9.84 | 6.87 | 7.02 |
| axon | 154 | 16 | 10.39 | 18.39 | 28.47 | 10.72 | 14.10 |
| microtubule cytoskeleton | 539 | 52 | 9.65 | 11.13 | 9.99 | 7.92 | 6.81 |
| actin cytoskeleton | 257 | 17 | 6.61 | 13.15 | 14.93 | 8.28 | 9.71 |
| extracellular matrix | 332 | 34 | 10.24 | 9.99 | 13.65 | 6.90 | 9.14 |

**Methodologies followed for the BMD calculations**

For the benchmark dose (BMD) calculation for thalidomide treatment, BMDExpress version 1.3 was used. The filtered 1026 transcripts from the F-statistics were used for BMD calculation, model selection and GO analysis in BMDExpress. The data were analysed using a one-way ANOVA. The filtered genes were fit with four statistical models (linear, second-degree polynomial, third-degree polynomial, and power models), and the least complex model was selected. The genes associated with the respective GO categories and the average BMD and benchmark dose lower confidence limit (BMDL) were determined for each GO classification.

**Benchmark dose assessment and GO analysis for the determination of mean BMD**

Traditional approaches to determine the lowest observed adverse effect levels (LOAEL) or no observed adverse effect levels (NOAEL) have limitations due to dose spacing and experimental sample size. BMD calculations from *in vitro* genomic data can overcome these constraints (1). BMD calculations integrate dose-response microarray data and GO classifications to predict the doses that perturb the respective functional GO categories. The genes from the BMD analysis were categorised based on the respective GO classifications. The average BMD dose was determined for the respective GO. The GO classification showed that mammary gland development is the most sensitive biological process (BP) at a concentration of 0.88 µM. The only clinical symptom for thalidomide-treated beagle dogs was mammary duct enlargement (2). Subsequently, skeletal system morphogenesis, limb development and embryonic skeletal system development GO categories were affected at the concentrations of 2.8, 3.04 and 3.1 µM, respectively. The BP heart development, urogenital system development, and striated muscle development were affected at concentrations of 6.13, 6.19 and 6.64 µM, respectively. The ectoderm related cellular components (CC) GO, such as dendrite, axon, and microtubule cytoskeleton-related genes, have mean BMD values of 9.7, 18.4 and 9.7 µM, respectively. Mesoderm-related CCs, such as actin cytoskeleton, extracellular matrix, and collagen, generate mean BMD values of 13, 10 and 13 µM, respectively.

**References**

(1) Filipsson AF, Sand S, Nilsson J, Victorin K. The benchmark dose method--review of available models, and recommendations for application in health risk assessment. Crit Rev Toxicol 2003; 33(5):505-542.

(2) Teo SK, Evans MG, Brockman MJ, Ehrhart J, Morgan JM, Stirling DI, Thomas SD. Safety profile of thalidomide after 53 weeks of oral administration in beagle dogs. Toxicol Sci 2001; 59(1):160-168.
